# Massive Emphysematous Pyelonephritis

**DOI:** 10.5811/cpcem.2016.11.32828

**Published:** 2017-01-18

**Authors:** Halsey Jakle, Adria Winter, Natalie Pena

**Affiliations:** Kern Medical, Department of Emergency Medicine, Bakersfield, California

## INTRODUCTION

A 58-year-old male presented to an outside hospital with altered mental status and right flank pain for three days. Septic work up, including computed tomography of the abdomen and pelvis, were significant for diabetic ketoacidosis, pyelonephritis, and significant air replacing much of the right kidney, consistent with emphysematous pyelonephritis (Image). The patient was transferred to our facility for a higher level of care.

The patient was stabilized, given intravenous (IV) antibiotics, and admitted to the intensive care unit with a diagnosis of septic shock secondary to emphysematous pyelonephritis.

## DISCUSSION

Our case presents an image of a condition that is rare and particularly severe, as shown by free air not only in the right renal parenchyma, but also extending outside the capsule, around the renal vasculature, and into the left peri-renal space.

Emphysematous pyelonephritis is a relatively rare infection, seen only 1–2 times per year in a typical busy urological department in the United States. It affects patients with diabetes in 95% of cases. *E. coli* and *Klebsiella* account for over 90% of cases, although *Proteus mirabilis*, *Pseudomonas*, and *Streptococcus* are also seen. Gas accumulates due to rapid necrosis of the renal parenchyma and peri-renal tissue, as opposed to gas appearing as a byproduct of anaerobic bacteria as is the case in necrotizing fasciitis. The condition is fatal if not treated appropriately, and the mainstay of treatment is nephrectomy in conjunction with IV antibiotics for severe, disseminated infection.

## Figures and Tables

**Image:**
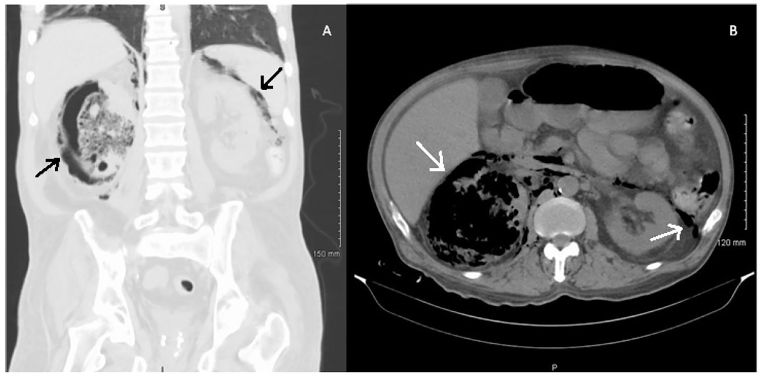
A. Coronal view of a computed tomography (CT) of the abdomen and pelvis, in the lung window, showing bilateral emphysema. B. Axial view of CT of the abdomen and pelvis, without contrast, showing emphysema replacing the right kidney.
